# Engineering Thermo-Responsive Hydrogels with Tailored Mechanics for Biomedical Integration

**DOI:** 10.3390/polym17172424

**Published:** 2025-09-08

**Authors:** Sungmo Choi, Minkyeong Pyo, Sangmin Lee, Yunseo Jeong, Yuri Nam, Seonghyeon Park, Yoon-A Jang, Kisung Kim, Chan Ho Park

**Affiliations:** Department of Chemical and Biological Engineering, Gachon University, Seongnam 13120, Republic of Korea; chltjdahah@gachon.ac.kr (S.C.); minrudxx5174@gachon.ac.kr (M.P.);

**Keywords:** poly(N-isopropylacrylamide), microneedle, thermo-responsive, mechanical strength, biomedical application

## Abstract

Poly(N-isopropylacrylamide) (PNIPAAm) hydrogels exhibit temperature-responsive volume changes near physiological temperature, but their low mechanical strength in the swollen state limits use in structurally demanding biomedical applications. In this study, we systematically investigated poly(NIPAAm-co-acrylamide), P(NIPAAm-co-AAm), hydrogels with varying AAm-to-NIPAAm ratios to explore the compositional trade-offs between thermal responsiveness and mechanical performance. Hydrogels were synthesized under fixed crosslinker and water content conditions, and evaluated through compressive mechanical testing, thermal swelling analysis, and crosslinking density estimation. Our results show that increasing AAm content enhances mechanical strength and stiffness but reduces the magnitude of temperature-induced volumetric shrinkage. An intermediate comonomer formulation demonstrated an optimal balance, maintaining both sufficient mechanical integrity for transdermal microneedle insertion and a reversible volume transition. This study highlights the potential of compositional tuning in hydrogel systems to meet the competing demands of responsiveness and durability in advanced biomedical applications.

## 1. Introduction

Thermally induced volumetric phase transitions, within the range of physiological temperatures, offer a uniquely biocompatible and controllable mechanism for biomedical applications such as localized drug delivery and minimally invasive diagnostics without the need for external energy sources such as lasers or electrical fields [1,2,3,4,5,6]. Poly(N-isopropylacrylamide), called PNIPAAm-based hydrogels, is widely recognized for their excellent thermo-responsiveness, exhibiting a distinct lower critical solution temperature (LCST) near physiological conditions (~32 °C) [7,8,9,10,11]. Above this transition temperature, PNIPAAm undergoes volumetric shrinkage, promoting enhanced drug release efficiency due to the expulsion of entrapped solvent [12,13]. However, PNIPAAm hydrogels exhibit extremely low mechanical strength. This mechanical weakness limits their applicability in biomedical systems that demand structural robustness under physiological mechanical stresses, such as implantable or wearable hydrogel-based devices, including microneedle (MN) systems [14,15,16]. MN systems, which utilize micron-scale needle structures to penetrate the skin barrier for efficient transdermal drug delivery, are particularly susceptible to mechanical failure during insertion or under sustained loading due to the inherently low strength of hydrogels [17].

To address this limitation, the copolymerization of other monomers that reinforce the mechanical properties, such as acrylates and vinyl monomers, including acrylamide (AAm) and vinyl sulfonic acid, has been proposed as a mechanical reinforcement strategy for the PNIPAAm matrix [15]. Comonomers typically introduce additional hydrogen bonding and network density, thereby significantly enhancing the mechanical integrity of the hydrogel [18]. However, this compositional modification inevitably compromises the sharp thermal transition behavior of PNIPAAm, leading to a partial loss of temperature sensitivity [19]. However, to the best of our knowledge, there has been no study that simultaneously analyzes the direct correlation between the thermo-responsivity and the mechanical properties, considering the various properties of soft matter (toe modulus, Young’s modulus, ultimate compressive stress, and modulus of resilience) of copolymerized PNIPAAm hydrogels, and further discusses their implications for biomedical applications.

Thus, our study aims to identify and optimize the balance between mechanical robustness and thermo-responsiveness in P(NIPAAm–co-AAm) hydrogels. By navigating this intrinsic trade-off, we seek to establish a design framework for hydrogel-based microneedles that can (i) reliably puncture the skin and (ii) maintain sufficient temperature-triggered drug release performance. This strategy is expected to enable more effective and minimally invasive transdermal delivery systems.

## 2. Materials and Methods

### 2.1. Materials

N-isopropylacrylamide stabilized with MEHQ (NIPAAm, ≥98.0%) was purchased from Tokyo Chemical Industry Co., Ltd. (Tokyo, Japan) Acrylamide (AAm, 98.5%) was obtained from Samchun Chemicals (Seoul, Korea). N,N′-Methylenebisacrylamide (MBAM, 99%), Ammonium persulfate (APS, ≥98%), N,N,N′,N′-tetramethylethylenediamine (TEMED, ~99%), and Rhodamine 6G were supplied by Sigma-Aldrich (St. Louis, MO, USA). Disposable plastic Petri dishes (diameter: 50 mm) were purchased from SPL Life Sciences (Pocheon, Republic of Korea). Porcine skin was purchased from Biozoa (Seoul, Republic of Korea). Deionized water (18 MΩ·cm) was used in all experiments.

### 2.2. Morphological and Mechanical Characterization

FT-IR (Vertex 70, Bruker, Billerica, MA, USA; Smart Materials Research Center for IoT supported by the Korea Basic Science Institute, NFEC-2007-12-049776) spectra of all samples were obtained in the range of 4000–700 cm^−1^ to analyze the chemical changes in the hydrogels according to the NIPAAm and AAm ratios. The samples were dried in a vacuum oven at 45 °C for 24 h prior to analysis. The internal microstructure and pore morphology were investigated using SEM (JSM-7500F, Jeol (Tokyo, Japan) and SU8600, Hitachi (Tokyo, Japan); Smart Materials Research Center for IoT supported by the Korea Basic Science Institute, NFEC-2023-02-285654). The samples were frozen in liquid nitrogen for 1 min immediately after fabrication, followed by freeze-drying for 48 h before imaging. To observe the tip morphology of the MNs, the samples were cut to expose their cross-sections and examined using an optical microscope, S16C (MICroscopes INC, St. Louis, MO, USA). The compressive strength of hydrogels with different compositions was measured using a UTM (Z010 TN, Zwick Roell (Ulm, Germany), and Instron Universal Testing Machine, 34SC-1 (Norwood, MA, USA); Smart Materials Research Center for IoT supported by the Korea Basic Science Institute, NFEC-2020-09-265008) with fully swollen samples. The samples were prepared in a cylindrical shape with an average diameter of 37.1 mm and a thickness of 3.6 mm, corresponding to a width-to-height ratio of approximately 1:10. The measurements were conducted at a crosshead speed of 1 mm/min using a 1 kN load cell. For each composition, three samples were tested.

### 2.3. Fabrication of Negative Molds

Negative molds for hydrogel fabrication were produced using a 3D printer (Photon Mono 4K, Anycubic, (Shenzhen, China)) with a clear, water-washable resin (Anycubic). Two types of molds were designed: cylindrical molds with an inner diameter of 30 mm, outer diameter of 40 mm, and height of 30 mm; and microneedle (MN) molds with an inner diameter of 6.58 mm, outer diameter of 8.58 mm, and height of 18 mm. The MN array consisted of 25 tips arranged in a 5 × 5 grid, with each tip featuring a square base of 0.73 mm × 0.73 mm and a height of 1.5 mm. After printing, the molds were washed with isopropyl alcohol (IPA) for 20 min and subsequently cured for 10 min. Both post-processing steps were performed using a Wash & Cure 2.0 machine (Anycubic, Shenzhen, China). The molds were air-dried at room temperature for 30 min. Subsequently, surface treatment was performed by immersing the molds in a 1 vol% solution of octadecyltrimethoxysilane in acetone. After 1 h of silanization, the molds were again dried at room temperature for an additional hour prior to use.

### 2.4. Preparation of P(NIPAAm-co-AAm)/PNIPAAm Hydrogels

P(NIPAAm-co-AAm) and PNIPAAm hydrogels were synthesized with varying monomer compositions as listed in Table 1. NIPAAm, AAm, and MBAM were mixed in a 20 mL vial at specific weight ratios. Subsequently, APS (100 mg/mL), Rhodamine 6G (10 mg/mL), and DI water were added to the vial. The hydrogel precursor solution was homogenized using a vortex mixer. A total of 3000 µL of the precursor was poured into the negative mold, followed by the addition of 7.5 µL of TEMED to initiate polymerization. After 15 min, the hydrogel samples were demolded and rinsed thoroughly with DI water before further use.

### 2.5. Temperature-Dependent Dimensional Analysis of Hydrogels

To examine the thermo-responsive dimensional behavior of the hydrogels, each sample was placed in a 50 mm plastic Petri dish filled with DI water. Four hydrogel samples, each with a distinct composition, and one control Petri dish (DI water only) were positioned on the hot plate. A digital thermometer was inserted into the control dish to monitor the water temperature, assuming thermal equilibrium with the other dishes. As the temperature was gradually increased over 30 min, photographs were taken at defined intervals. The dimensional changes in hydrogel area were quantified using ImageJ (ImageJ 1.54g) software.

### 2.6. Skin Penetration Test of P(NIPAAm-AAm)/P(NIPAAm) Microneedles

Porcine skin was cut into 3 cm × 3 cm sections and washed three times with a 1:1 volume ratio of ethanol–water solution. The microneedles were dried at room temperature for 24 h, placed onto the porcine skin, and covered with an acrylic plate to ensure uniform load distribution. A 1 kg weight was then applied for 3 s to assess skin penetration.

## 3. Results and Discussion

**Figure 1:**
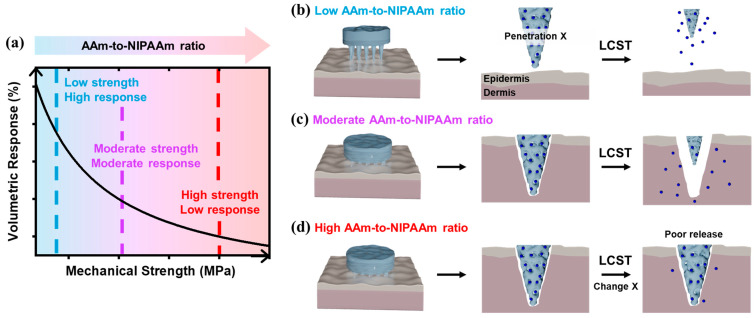
Schematic illustration of the mechanical tunability of temperature-responsive hydrogels. (**a**) Trade-off behavior between mechanical strength and thermo-responsiveness in PNIPAAm-based hydrogel depending on the AAm-to-NIPAAm ratio. P(NIPAAm-co-AAm) hydrogel-based MNs with (**b**) low content of AAm, (**c**) moderate content of AAm, and (**d**) high content of AAm in PNIPAAm hydrogel for temperature-responsive drug release as a form of MN penetrating in skin dermis.

To investigate the balance between mechanical strength and thermo-responsiveness in hydrogel, we systematically varied the ratio of NIPAAm and AAm monomers (Figure 1). By adjusting the relative content of these two monomers, we aimed to identify an optimal formulation that ensures both sufficient skin-penetrating mechanical strength and thermo-responsive volumetric shrinkage upon thermal stimulation. As a proof-of-concept, P(NIPAAm-co-AAm) hydrogels were synthesized by copolymerizing AAm with NIPAAm to systematically investigate the trade-off between mechanical reinforcement and temperature-responsive volumetric behavior [18]. As shown in Figure 1a, increasing the AAm-to-NIPAAm ratio enhances the mechanical strength of the hydrogel network, whereas the thermo-responsive volumetric change in the hydrogel is diminished. This compositional dependency illustrates a critical balance between structural robustness and stimulus-responsiveness (Figure 1b–d). Hydrogels with a low AAm-to-NIPAAm ratio exhibit drastic thermo-responsive volumetric changes upon temperature variation; however, their mechanical property remain insufficient for applications requiring structural robustness, such as skin piercing capability in MN system, as illustrated in Figure 1b. In contrast, hydrogels synthesized with a high AAm-to-NIPAAm ratio achieve enhanced mechanical strength but display minimal or negligible temperature responsiveness (Figure 1d). As demonstrated in Figure 1c, a moderately balanced copolymer composition enables both adequate mechanical properties and reversible temperature-induced volumetric transitions, underscoring the importance of compositional tuning in designing functional hydrogels.

Table 1 summarizes the material compositions of the four hydrogel formulations used in this study. To systematically investigate the influence of comonomer composition on the thermo-responsive behavior and mechanical properties of the resulting hydrogels, the samples were designated according to their monomeric feed ratios as follows: 1.00:0.00, 0.66:0.33, 0.50:0.50, and 0.33:0.66 (NIPAAm:AAm). Each formulation maintained a constant total monomer and crosslinker concentration to ensure comparability across samples. The fluorescent tracer (Rhodamine 6G) was used for visual guidance. This series of hydrogels enables a controlled investigation of how increasing AAm content enhances mechanical properties at the expense of LCST-driven responsiveness, guiding an effective design strategy for MN applications.

**Figure 2:**
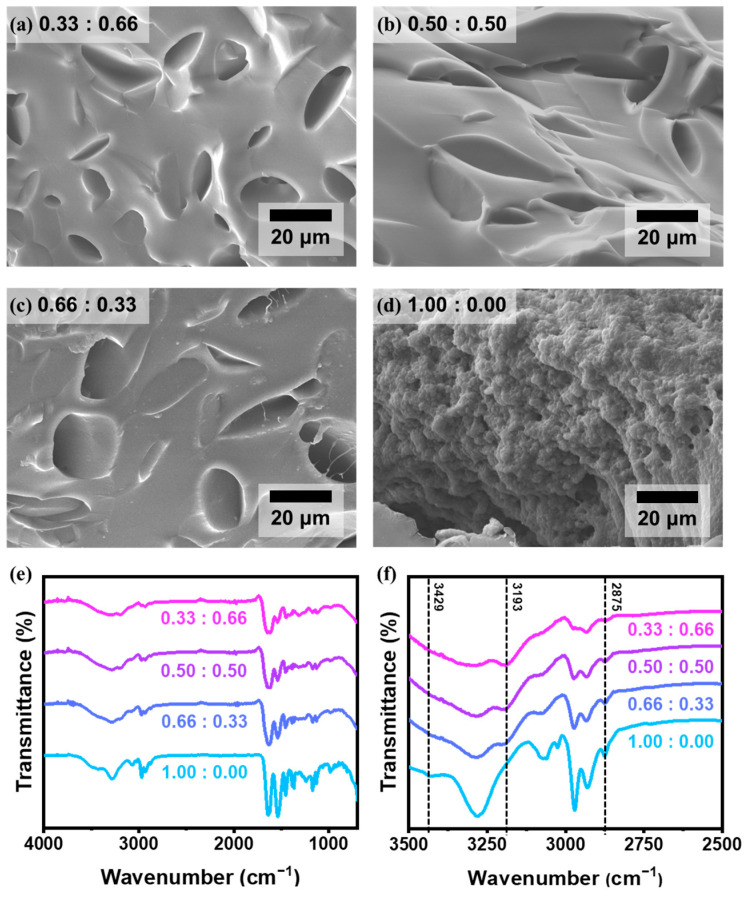
SEM images of the cross-sectional views of (**a**) 0.33:0.66, (**b**) 0.50:0.50, (**c**) 0.66:0.33, and (**d**) 1.00:0.00. (**e**,**f**) FT-IR spectra of P(NIPAAm-co-AAm) hydrogels with different monomer ratios. (**e**) Full-range spectra (4000–700 cm^−1^) showing overall vibrational band profiles of the hydrogel networks. (**f**) Enlarged view of the 3500–2500 cm^−1^ region highlighting key N–H and C–H stretching vibrations.

We investigated the cross-sectioned surface of hydrogels of 0.33:0.66, 0.50:0.50, 0.66:0.33, and 1.00:0.00 using SEM (Figure 2a–d). Hydrogels containing AAm—specifically the 0.33:0.66, 0.50:0.50, and 0.66:0.33—exhibited comparable morphologies characterized by micron-scale porous structures. In contrast, the 1.00:0.00 sample, composed of NIPAAm without AAm monomer, revealed a markedly different morphology with nanopores and distinct spherical globule-like morphology, indicative of a phase-separated microstructure unique to homopolymeric PNIPAAm networks. In addition, FT-IR analysis was performed to confirm the chemical structure and compositional variation in the synthesized P(NIPAAm-co-AAm) hydrogels (Figure 2e,f). The spectra reveal characteristic vibrational bands that correlate with the monomer composition, particularly in the 3500–2500 cm^−1^ region. A distinct absorption band observed at 3429 cm^−1^ corresponds to the N–H stretching vibration of secondary amide groups originating from NIPAAm units (Table S1) [20]. As the proportion of AAm increases, the intensity of this peak decreases, indicating a relative dilution of NIPAAm-derived secondary amides within the hydrogel network. Conversely, a shoulder peak appears at 3193 cm^−1^, which is attributed to the N–H symmetric stretching of primary amide groups present in AAm. This peak is absent in the pure PNIPAAm sample (1.00:0.00) but becomes increasingly prominent as the AAm content rises, providing spectroscopic evidence of successful copolymerization and incorporation of acrylamide monomers. Additionally, the band near 2875 cm^−1^ corresponds to C–H stretching vibrations, primarily from the isopropyl groups of NIPAAm [21]. A gradual attenuation of this peak is observed with increasing AAm content, further corroborating the decreasing fraction of NIPAAm units. Collectively, these FT-IR results confirm the progressive incorporation of AAm and corresponding compositional variation across the hydrogel series, validating the intended tuning of copolymer networks.

**Figure 3:**
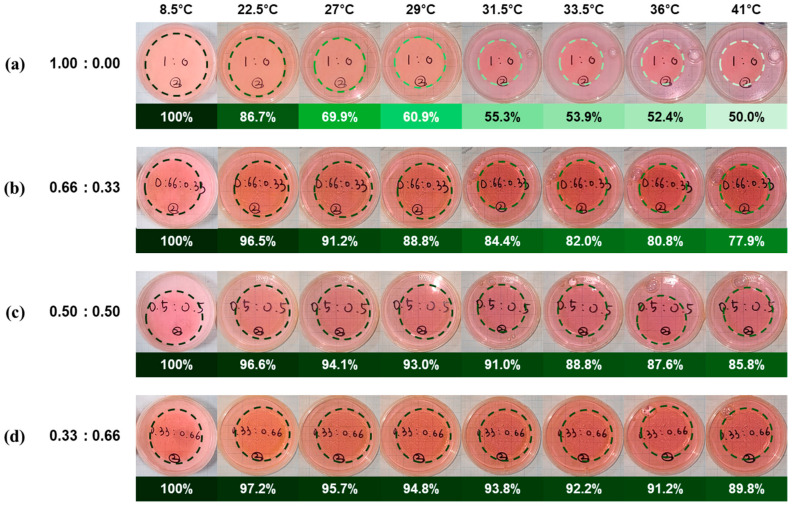
Volumetric changes in P(NIPAAm-co-AAm) hydrogels with varying monomer ratios—(**a**) 1.00:0.00, (**b**) 0.66:0.33, (**c**) 0.50:0.50, and (**d**) 0.33:0.66—measured in water across a temperature range of 8.5 to 41 °C.

**Figure 4:**
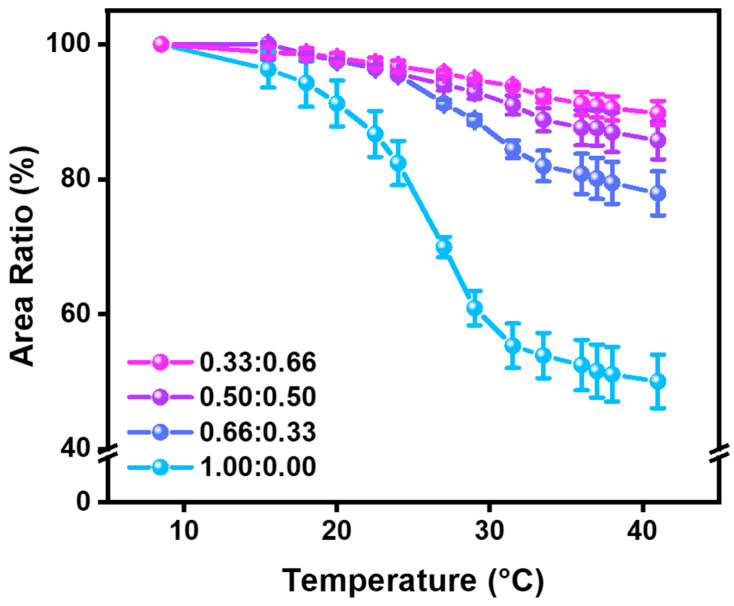
The area ratio curves of the hydrogels in water at different temperatures.

To assess the thermo-responsive behavior of the hydrogels, their dimensional changes were analyzed across a temperature range of 8.5 °C to 41 °C. The area of each hydrogel was measured from top-view images (Figure 3), and the values were normalized to the initial area measured at 8.5 °C, assuming fully swollen equilibrium at that temperature. The digitally converted areal data from Figure 3 are listed in Table S2 and plotted in Figure S1, and the normalized area changes are listed in Table S3 and plotted in Figure 4. All hydrogel compositions exhibited a temperature-dependent decrease in area, indicating that thermal shrinkage is characteristic of LCST-type phase transitions. The 1.00:0.00 formulation (pure PNIPAAm) showed a sharp decrease beginning near 22.5 °C, with a reduction of more than 10% occurring between 20 °C and 24 °C. The area ratio dropped to 0.5530 at 31.5 °C and plateaued at ~0.51 above 36 °C, consistent with PNIPAAm’s known LCST near 32 °C. This represents an approximate 50% reduction in surface area relative to the fully swollen state, which corresponds to roughly a 65% decrease in volume assuming isotropic shrinkage. In contrast, hybrid hydrogels with higher AAm content demonstrated more moderate and gradual transitions. The 0.33:0.66 hydrogel began decreasing by more than 10% only after 29 °C, reaching 0.9050 at 41 °C—a total area reduction of ~10% (a 15% decrease in volume), much lower than that of the pure PNIPAAm. Similarly, the 0.50:0.50 and 0.66:0.33 compositions reached saturation around 36 °C with final area ratios of 0.8695 (a 19% decrease in volume) and 0.7789 (a 31% decrease in volume), respectively. These results confirm that increasing AAm content suppresses the thermal responsiveness of the hydrogel network, elevating LCST and dampening the volume phase transition. The transition sharpness and amplitude were most pronounced in the 1.00:0.00 hydrogel, while AAm-rich formulations showed broader, less steep shrinkage profiles. These observations support the hypothesis that the hydrophilic and non-thermo-responsive nature of AAm interferes with the cooperative chain collapse associated with the LCST transition of PNIPAAm. AAm is a relatively hydrophilic comonomer that introduces additional hydrogen-bonding sites within the hydrogel network, thereby enhancing its overall water affinity. Incorporation of AAm leads to an upward shift in the LCST, while simultaneously broadening the volume phase transition. Consequently, higher AAm content results in a more gradual thermal response with reduced volumetric shrinkage, which mitigates the chain collapse typically observed in pure PNIPAAm systems. This behavior indicates that the hydrophilic and non-thermo-responsive nature of AAm disrupts the cooperative dehydration and intrachain association of PNIPAAm, thereby providing a means to modulate the trade-off between mechanical reinforcement and thermo-responsiveness. In addition, these volumetric responses of hydrogels were reversely operated under thermal transition cycles (8.5↔41 °C) as shown in Figure S2.

**Figure 5:**
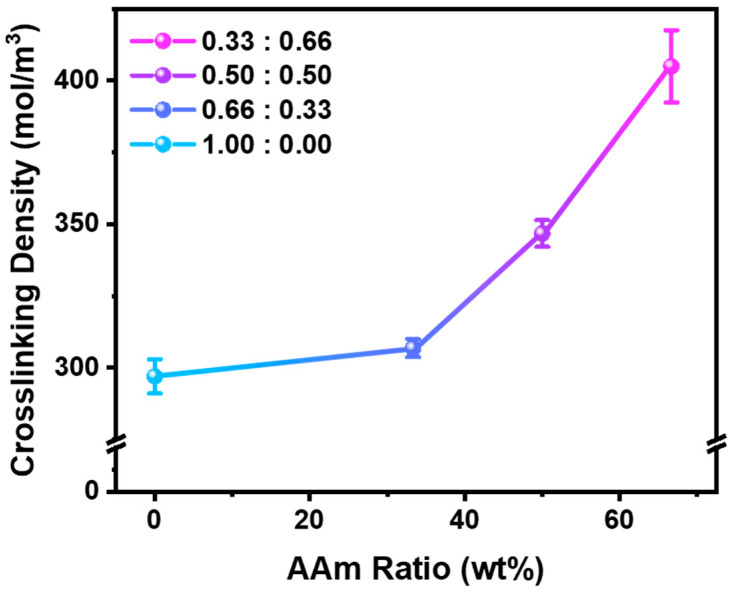
Crosslinking density of P(NIPAAm-co-AAm) hydrogels with different monomer ratios.

Based on the data presented in Table S4 and Figure 5, we quantitatively investigated the relationship between monomer composition and crosslinking density to gain deeper insight into the observed differences in temperature-induced volumetric behavior. Using the Flory–Rehner Equation (1) [22,23], we calculated the crosslinking density (N) of hydrogels with varying AAm:NIPAM ratios by comparing their equilibrium swollen and dried states.

(1) $$N=-\frac{ln\left(1-v_{h}\right)+v_{h}+\chi_{copolymer}v_{h}^{2}}{V_{w}\left({v_{h}}^{1/3}-\frac{v_{h}}{2}\right)}$$

(2) $$\chi_{copolymer}=\sum_{i}f_{i}\chi_{i}$$

(3) $$v_{h}=\frac{m_{d}/\rho_{copolymer}}{{(m}_{d}/\rho_{copolymer})+(m_{s}-m_{d})/\rho_{w}}$$

(4) $$\rho_{copolymer}=\sum_{i}f_{i}\rho_{i}$$

where $v_{h}$ is the polymer volume fraction in the swollen hydrogel, χ is the Flory–Huggins polymer–solvent interaction parameter, $f_{i}$ is the volume fraction of polymer component *i*, $V_{w}$ is the molar volume of water, $m_{d}$ is the dry gel weight, $\rho_{i}$ is the density of polymer component *i*,$m_{s}$ is the swollen gel weight, $\rho_{w}$ is the density of water. The Flory–Rehner equation, while not fully accounting for the high water content, non-ideal network structures, defects, and ionic effects inherent to hydrogels, was applied in this study as a framework to compare relative trends among different formulations. The results show a clear trend of increasing crosslinking density as the AAm content increases, ranging from 297 ± 5 mol/m^3^ for 1.00:0.00 to 405 ± 12 mol/m^3^ for 0.33:0.66 compositions. This increase in network density corresponds well with the diminished volume phase transition behavior observed in hydrogels with higher AAm content, supporting the notion that enhanced crosslinking restricts polymer chain mobility and limits water uptake. Furthermore, this trend can be aligned with the mechanical reinforcement effects, indicating that AAm incorporation not only stiffens the network but also modulates its thermo-responsive swelling characteristics.

**Figure 6:**
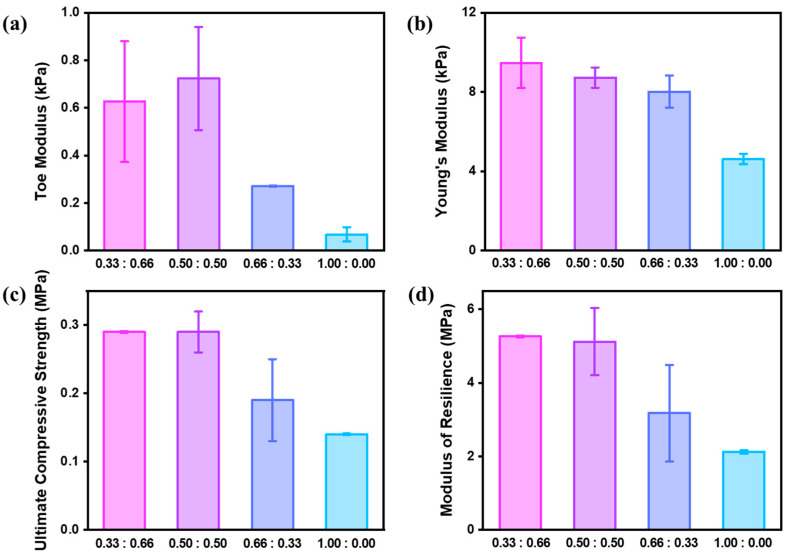
Mechanical properties of P(NIPAAm-co-AAm) hydrogels with varying monomer ratios, determined from compressive stress–strain measurements: (**a**) toe modulus, (**b**) Young’s modulus, (**c**) ultimate compressive stress, and (**d**) modulus of resilience.

To comprehensively assess the mechanical behavior of the swollen P(NIPAAm-co-AAm) hydrogels, we conducted the compression test using a Universal Testing Machine (Figure S3). Figure S4 presents the stress–strain curves for all hydrogel formulations subjected to uniaxial compression. We quantified four representative parameters derived from compressive stress–strain curves: toe modulus, Young’s modulus (heel region), ultimate compressive strength, and modulus of resilience (Figure 6 and Table S5). These parameters provide critical insights into the mechanical response of gel-like or soft matter systems, particularly under physiologically relevant loads [24,25]. The toe modulus, indicative of the initial compliance under minimal stress, showed a marked increase with AAm incorporation. The pure PNIPAAm hydrogel (1.00:0.00) exhibited a toe modulus of 0.07 kPa, whereas the 0.66:0.33, 0.50:0.50 and 0.33:0.66 hydrogels exhibited significantly enhanced values of 0.27, 0.72 and 0.63 kPa, respectively, reflecting increased initial stiffness. Similarly, the Young’s modulus, reflecting overall stiffness in the linear deformation region, increased consistently from 4.62 kPa for 1.00:0.00 to 9.47 kPa for 0.33:0.66, confirming the AAm-induced reinforcement of the polymer network. The ultimate compressive strength, which captures the maximum strength of the hydrogel under compressive load, also improved with increasing AAm content. Hydrogels with higher AAm ratios (0.50:0.50 and 0.33:0.66) demonstrated strengths of approximately 0.29 MPa, more than double that of the PNIPAAm-only hydrogel (0.14 MPa). The modulus of resilience, representing the energy absorbed before yielding, further emphasized this enhancement trend, rising from 2.12 MPa (1.00:0.00) to 5.27 MPa (0.33:0.66), indicating a substantial improvement in mechanical robustness. While each parameter exhibits unique sensitivity to composition, the overall trend demonstrates an increase in stiffness and strength, attributable to the denser and more rigid polymer network introduced by acrylamide comonomer incorporation. As the AAm content increased from 0.00 to 0.50, the hydrogels exhibited gradual improvements in toe modulus, Young’s modulus, ultimate compressive stress, and modulus of resilience. Beyond this composition, the mechanical properties reached a relatively saturated state, representing the characteristics of high-AAm-content hydrogels. A similar trend was observed in the volumetric transition behavior, where the extent of swelling gradually decreased from the 1.00:0.00 to the 0.50:0.50 formulation, and subsequently showed little further change (Figure 4). Taken together, we suggest defining the formulations as follows: AAm content from 0 to 10 wt% (4 mol%) represents a low AAm-to-NIPAAm ratio, 10 to 50 wt% (20 mol%) corresponds to an intermediate ratio, and above 50 wt% is categorized as a high ratio. These results demonstrate that the incorporation of AAm enhances the mechanical robustness of PNIPAAm hydrogels, broadening their utility in diverse biomedical applications such as implantable and wearable systems.

**Figure 7:**
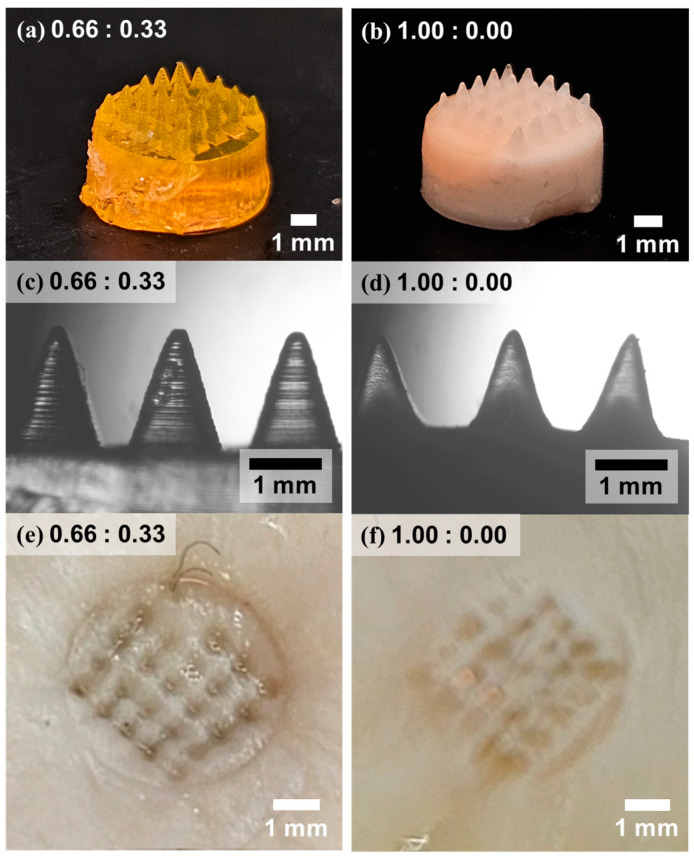
Photographic and optical microscopic images of PNIPAAm hydrogel-based MNs fabricated with different monomer ratios. (**a**,**b**) Macroscopic images of MN arrays synthesized with 0.66:0.33 and 1.00:0.00 (NIPAAm:AAm) ratios, respectively. (**c**,**d**) Optical microscope images showing the geometry and integrity of the corresponding MNs. (**e**,**f**) Photographic evidence of puncture marks on porcine skin following insertion of MNs with 0.66:0.33 and 1.00:0.00, respectively.

MN arrays are presented as a proof-of-concept application, where sufficient load-bearing capacity and dimensional stability are critical for reliable skin penetration [26,27]. To evaluate the influence of AAm incorporation on MN fabrication and performance, hydrogel-based MN arrays were prepared using two formulations: 0.66:0.33 and 1.00:0.00 (NIPAAm:AAm). Master molds with negative needle cavities were fabricated via digital light processing-based additive manufacturing, and hydrogel precursors were cast into these molds, polymerized, and subsequently peeled off to yield MN patches. As shown in Figure 7a,b, both formulations successfully formed hydrogel-based MN arrays; however, notable differences in structural fidelity were observed. The 0.66:0.33 sample exhibited well-defined, sharply contoured needles with higher transparency, while the 1.00:0.00 sample presented less distinct needle shapes, with a number of malformed or incompletely formed tips and a generally opaque appearance. Optical microscopy images in Figure 7c,d further highlight the morphological distinctions: the 0.66:0.33 sample preserved microscale features of the layered 3D printed mold, whereas such fine details were not retained in the 1.00:0.00 counterpart, indicating lower pattern fidelity due to insufficient mechanical integrity. The practical consequence of these structural differences was evident during porcine skin insertion experiments (Figure 7e,f). The 0.66:0.33 MN patch produced clearly defined puncture marks consistent with the original needle geometry, while the 1.00:0.00 patch left irregular and shallow penetration marks. In some cases, individual needles fractured and remained embedded in the tissue, highlighting the insufficient mechanical strength of the only PNIPAAm formulation. In addition, Figure S5 compares the penetration depth of MNs into artificial skin (parafilm) [28]. Consistent with the mechanical strength results and the observations in Figure 7, a clear trend was observed in which higher AAm content enabled deeper and more stable penetration. The average penetration depth increased from 317 µm for pure PNIPAAm (1.00:0.00) to 584 µm at an AAm:PNIPAAm ratio of 0.33:0.66. These findings confirm that a modest incorporation of AAm significantly enhances the mechanical robustness necessary for successful skin penetration, without compromising needle resolution. According to reported measurements on human skin, the Young’s modulus ranges from 2.9 to 150 MPa depending on the donor site [29,30]. Based on this, for hydrogels with a high content of AAm, a resistive force of 0.29 MPa necessitates a tip diameter of 36–260 μm, whereas a reduced resistive force of 0.14 MPa in pure PNIPAAm hydrogels corresponds to a smaller tip diameter of 25–181 μm. These dimensional requirements approach the resolution limits of 3D printing technologies (e.g., Mono 4K, 3840 × 2400 resolution, 35 μm XY pixel size). This finding highlights that enhancing the mechanical robustness of responsive hydrogels would expand the design versatility and practical feasibility of microneedle arrays [31,32]. To this end, we propose that an optimal balance can be achieved by concurrently tailoring the required drug dose and the concentration of the hydrogel reservoir. This suggests the feasibility of tailoring MN performance by modulating the AAm-to-NIPAAm ratio to match drug delivery requirements [33,34]. For instance, drug concentration and release volume may be coordinated with the hydrogel’s volumetric transition and mechanical threshold to optimize delivery efficiency [35].

## 4. Conclusions

In this study, we systematically investigated the relationship between thermo-responsive behavior and mechanical performance in PNIPAAm-based hydrogels modified with AAm comonomers. By tuning the AAm-to-NIPAAm ratio, we demonstrated that a desirable compromise between thermo-responsive volumetric change and mechanical robustness can be achieved within a single hydrogel system. The resulting P(NIPAAm-co-AAm) hydrogels exhibited sufficient stiffness (Young’s modulus 8–9 kPa) to successfully penetrate porcine skin, while still retaining a temperature-induced volumetric shrinkage of approximately 15–31%, confirming their applicability as MN array platforms. In contrast, hydrogels composed solely of NIPAAm underwent significant temperature-induced volume reduction (~65%) but lacked the mechanical strength (Young’s modulus ~4 kPa) required for transdermal insertion, thereby limiting their practical utility. The incorporation of AAm increased crosslinking density (up to ~405 mol/m^3^) and altered internal microstructures, contributing to improved load-bearing capacity and deformation resistance. Our results show that adjusting the comonomer ratio allows for controlled tuning of thermal responsiveness and mechanical strength in stimuli-responsive hydrogels. Such compositional tuning opens new opportunities for application-specific optimization in transdermal delivery, implantable devices, and smart biomaterials [36,37]. Future research can use this strategy to design hydrogel systems optimized for controlled drug release and actuation, supporting broader use in personalized biomedical devices [6,38,39,40,41].

## Figures and Tables

**Table 1:** Material compositions of P(NIPAAm-co-AAm) hydrogels.

Materials	Sample Notation			
	1.00:0.00	0.66:0.33	0.50:0.50	0.33:0.66
NIPAAm (mg)	870	580	435	290
AAm (mg)	0	290	435	580
MBAM (mg)	60			
APS (100mg/mL) (μL)	100			
TEMED (μL)	7.5			
Rhodamine 6G (mg)	0.5			
DI Water (μL)	2700			

## Data Availability

The raw data supporting the conclusions of this article will be made available by the authors on request.

## Supplementary Materials

The following supporting information can be downloaded at https://www.mdpi.com/article/10.3390/polym17172424/s1, Table S1. Table of peak assignments of FT-IR spectra. Table S2. The area of P(NIPAAm-co-AAm) Hydrogels in DI water at different temperatures. Table S3. The area ratio of P(NIPAAm-co-AAm) hydrogels in DI water at different temperatures. Table S4. Average crosslinking density of hydrogels with different monomer ratios. Table S5. Average values of the mechanical properties of P(NIPAAm-co-AAm) hydrogels calculated from compressive stress–strain curves. Figure S1. The area curves of the hydrogels in DI water at different temperatures. Figure S2. Reversibility test of the volumetric changes in swollen (8.5 °C) and shrunken state (41 °C) of hydrogels varying the content of AAm. Figure S3. Images of the (a) PNIPAAm and (b) P(NIPAAm-co-AAm) hydrogels in maximum swelling states during the compression test. Figure S4. Stress–strain curves for all hydrogel formulations subjected to uniaxial compression. Figure S5. Cross-sectional digital images of microneedles after penetration into artificial skin and average penetration depth.
