# Supplementary material for: Engineering Thermo-Responsive Hydrogels with Tailored Mechanics for Biomedical Integration
Source: Polymers (Basel). 2025 Sep 8;17(17):2424. doi: 10.3390/polym17172424 (PMC12431455; doi:10.3390/polym17172424)
Supplement: Supplementary file 1 [file polymers-17-02424-s001.zip › polymers-3827533-supplementary.pdf]

Supporting Information

Engineering Thermo-Responsive Hydrogels with Tailored Mechanics for Biomedical Integration

Sungmo Choi †, Minkyeong Pyo †, Sangmin Lee †, Yunseo Jeong, Yuri Nam, Seonghyeon Park, Yoon-A Jang, Kisung Kim and Chan Ho Park *

Department of Chemical and Biological Engineering, Gachon University, Seongnam 13120, Republic of Korea
* Correspondence: chhopark@gachon.ac.kr
† These authors contributed equally to this work.

Table of Contents

1. Supplementary Tables

- Table of peak assignments of FT-IR spectra S1
- The area of P(NIPAAm-co-AAm) Hydrogels in DI water at different temperatures S2
- The area ratio of P(NIPAAm-co-AAm) hydrogels in DI water at different temperatures S3
- Average crosslinking density of hydrogels with different monomer ratios S4
- Average values of the mechanical properties of P(NIPAAm-co-AAm) hydrogels calculated from compressive stress-strain curves S5

2. Supplementary Figures

- The area curves of the hydrogels in DI water at different temperatures S1
- Reversibility test of the volumetric changes in swollen (8.5 °C) and shrunken state (41 °C) of hydrogels varying the content of AAm. S2
- Images of the a) PNIPAAm and b) P(NIPAAm-co-AAm) hydrogels in maximum swelling states during the compression test S3
- Stress–strain curves obtained from three independent replicates for all hydrogel formulations under uniaxial compression: (a) 0.33:0.66, (b) 0.50:0.50, (c) 0.66:0.33, and (d) 1.00:0.00. S4
- Cross-sectional digital images of microneedles after penetration into artificial skin (parafilm) for formulations with PNIPAAm:AAm ratios of a) 0.33:0.66, b) 0.50:0.50, c) 0.66:0.33, and d) 1.00:0.00. Average penetration depth of microneedles into artificial skin (parafilm) for formulations with PNIPAAm:AAm ratios of 0.33:0.66, 0.50:0.50, 0.66:0.33, and 1.00:0.00. S5

Table S1. Table of peak assignments of FT-IR spectra.

**Wavenumber (cm^{-1})**	**Assignment**
3429	N-H stretching of secondary amide
3193	N-H stretching of primary amide
2875	C-H stretching

Table S2. The area of P(NIPAAm-co-AAm) Hydrogels in DI water at different temperatures.

Temperature (°C)	Area (cm^2)			
	**1.00 : 0.00**	**0.66 : 0.33**	**0.50 : 0.50**	**0.33 : 0.66**
8.5	14.77 ± 0.18	12.75 ± 0.34	11.91 ± 0.20	11.22 ± 0.14
15.5	14.23 ± 0.49	12.60 ± 0.40	11.91 ± 0.17	11.09 ± 0.20
18.0	13.94 ± 0.59	12.56 ± 0.38	11.74 ± 0.21	11.07 ± 0.20
20.0	13.49 ± 0.57	12.43 ± 0.36	11.65 ± 0.20	10.99 ± 0.20
22.5	12.82 ± 0.56	12.30 ± 0.33	11.50 ± 0.20	10.91 ± 0.20
24.0	12.18 ± 0.53	12.17 ± 0.32	11.39 ± 0.23	10.86 ± 0.21
27.0	10.33 ± 0.29	11.63 ± 0.28	11.20 ± 0.24	10.74 ± 0.17
29.0	9.00 ± 0.41	11.31 ± 0.26	11.07 ± 0.19	10.63 ± 0.13
31.5	8.18 ± 0.49	10.76 ± 0.33	10.84 ± 0.24	10.52 ± 0.12
33.5	7.97 ± 0.49	10.45 ± 0.42	10.58 ± 0.31	10.35 ± 0.15
36.0	7.76 ± 0.53	10.30 ± 0.45	10.45 ± 0.40	10.23 ± 0.19
37.0	7.63 ± 0.55	10.22 ± 0.45	10.44 ± 0.40	10.19 ± 0.20
38.0	7.56 ± 0.56	10.13 ± 0.46	10.37 ± 0.43	10.15 ± 0.20
41.0	7.40 ± 0.55	9.94 ± 0.49	10.22 ± 0.42	10.07 ± 0.21

Table S3. The area ratio of P(NIPAAm-co-AAm) hydrogels in DI water at different temperatures.

Temperature (°C)	Area (cm^2)			
	**1.00 : 0.00**	**0.66 : 0.33**	**0.50 : 0.50**	**0.33 : 0.66**
8.5	100.00 ± 0.00	100.00 ± 0.00	100.00 ± 0.00	100.00 ± 0.00
15.5	96.31 ± 2.69	98.82 ± 1.06	100.0 ± 0.31	98.84 ± 0.72
18.0	94.29 ± 3.55	98.49 ± 0.95	98.58 ± 0.16	98.68 ± 0.72
20.0	91.23 ± 3.41	97.51 ± 0.34	97.81 ± 0.07	97.96 ± 0.74
22.5	86.71 ± 3.40	96.46 ± 0.18	96.61 ± 0.27	97.25 ± 0.79
24.0	82.40 ± 3.28	95.45 ± 0.04	95.66 ± 0.19	96.77 ± 0.83
27.0	69.93 ± 1.15	91.22 ± 0.59	94.05 ± 0.86	95.72 ± 0.42
29.0	60.89 ± 2.56	88.79 ± 0.74	92.96 ± 1.01	94.81 ± 0.03
31.5	55.30 ± 3.31	84.44 ± 1.29	91.00 ± 1.41	93.79 ± 0.17
33.5	53.86 ± 3.36	81.96 ± 2.28	88.81 ± 1.73	92.25 ± 0.90
36.0	52.44 ± 3.74	80.79 ± 2.99	87.64 ± 2.06	91.24 ± 1.73
37.0	51.56 ± 3.95	80.11 ± 3.05	87.58 ± 2.59	90.88 ± 1.75
38.0	51.08 ± 4.05	79.47 ± 3.12	87.58 ± 2.95	90.88 ± 1.80
41.0	50.02 ± 3.96	77.89 ± 3.26	86.95 ± 2.82	90.50 ± 1.80

Table S4. Average crosslinking density of hydrogels with different monomer ratios.

Sample	Crosslinking Density (mol/m^3)			
	**1.00 : 0.00**	**0.66 : 0.33**	**0.50 : 0.50**	**0.33 : 0.66**
1	318.4	403.4	445.8	581.1
2	335.7	405.9	467.8	470.7
3	306.5	389.3	463.3	433.8
4	330.8	398.5	447.3	477.6
5	361.0	389.9	438.8	495.7
6	347.6	393.7	425.7	472.8
7	346.9	390.2	467.9	471.7
8	366.9	369.4	472.4	483.1
Average	339.2 ± 7.3	392.5 ± 4.0	453.6 ± 6.0	485.8 ± 15.0

Table S5. Average values of the mechanical properties of P(NIPAAm-co-AAm) hydrogels calculated from compressive stress-strain curves.

		**1.00 : 0.00**	**0.66 : 0.33**	**0.50 : 0.50**	**0.33 : 0.66**
**Toe Modulus (kPa)**	**1**	0.01	0.27	0.2	0.6
	**2**	0.06	0.27	1.05	0.69
	**3**	0.14	0.28	0.92	1.13
	**Average**	0.07 ± 0.03	0.27 ± 0.00	0.72 ± 0.22	0.63 ± 0.25
**Young's Modulus (kPa)**	**1**	4.60	9.94	9.68	12.33
	**2**	5.18	7.52	7.54	9.10
	**3**	4.07	6.59	8.98	6.99
	**Average**	4.62 ± 0.26	8.02 ± 0.82	8.73 ± 0.51	9.47 ± 1.27
**Ultimate Compressive Strength (MPa)**	**1**	0.14	0.07	0.35	0.29
	**2**	0.15	0.23	0.24	0.28
	**3**	0.14	0.26	0.28	0.29
	**Average**	0.14 ± 0.00	0.19 ± 0.06	0.29 ± 0.03	0.29 ± 0.00
**Modulus of Resilience (MPa)**	**1**	2.04	0.76	6.94	5.27
	**2**	2.18	3.44	4.33	5.23
	**3**	2.13	5.31	4.09	5.30
	**Average**	2.12 ± 0.04	3.17 ± 1.32	5.12 ± 0.91	5.27 ± 0.02

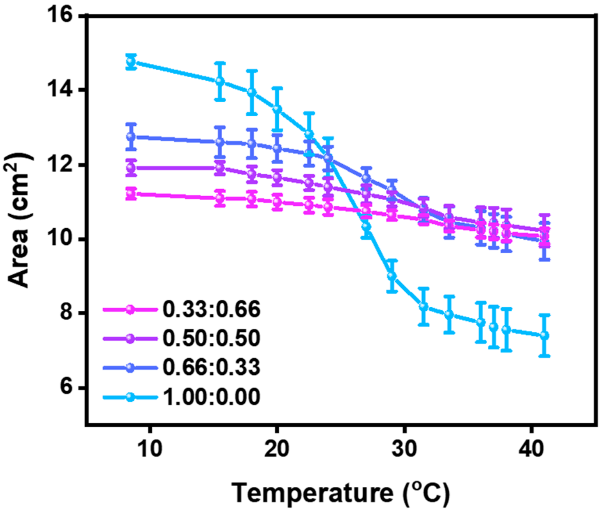

Figure S1. The area curves of the hydrogels in DI water at different temperatures.

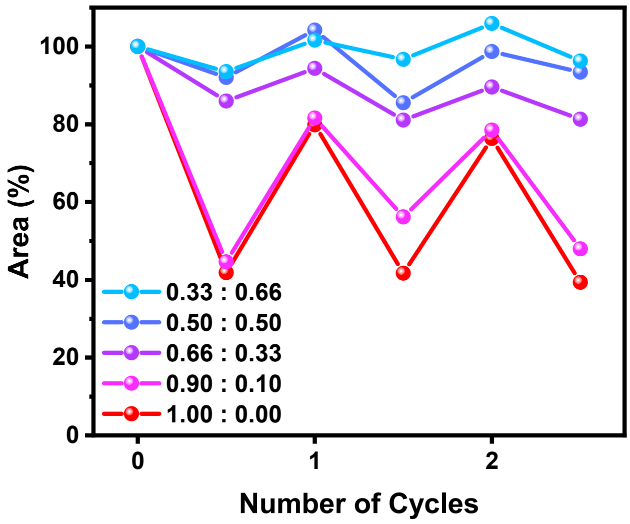

Figure S2. Reversibility test of the volumetric changes in swollen (8.5 ℃) and shrunken state (41 ℃) of hydrogels varying the content of AAm.

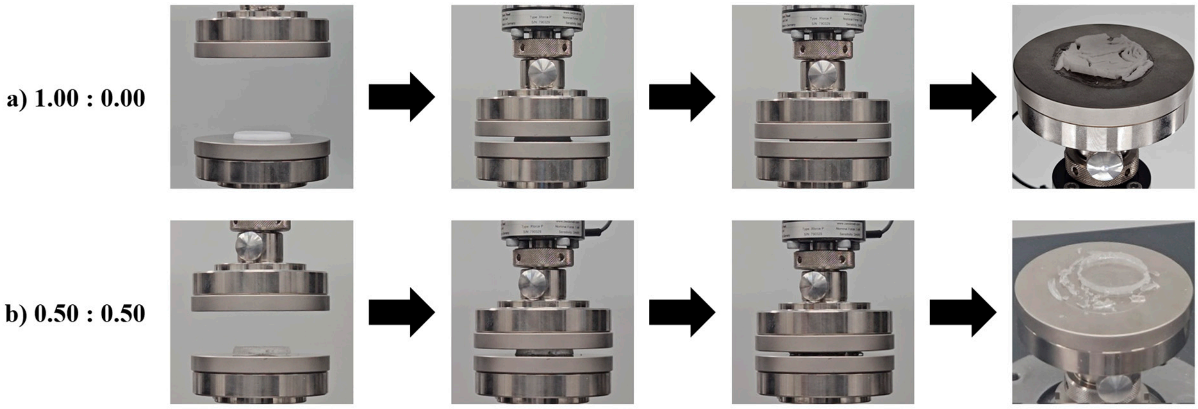

Figure S3. Images of the a) PNIPAAm and b) P(NIPAAm-co-AAm) hydrogels in maximum swelling states during the compression test.

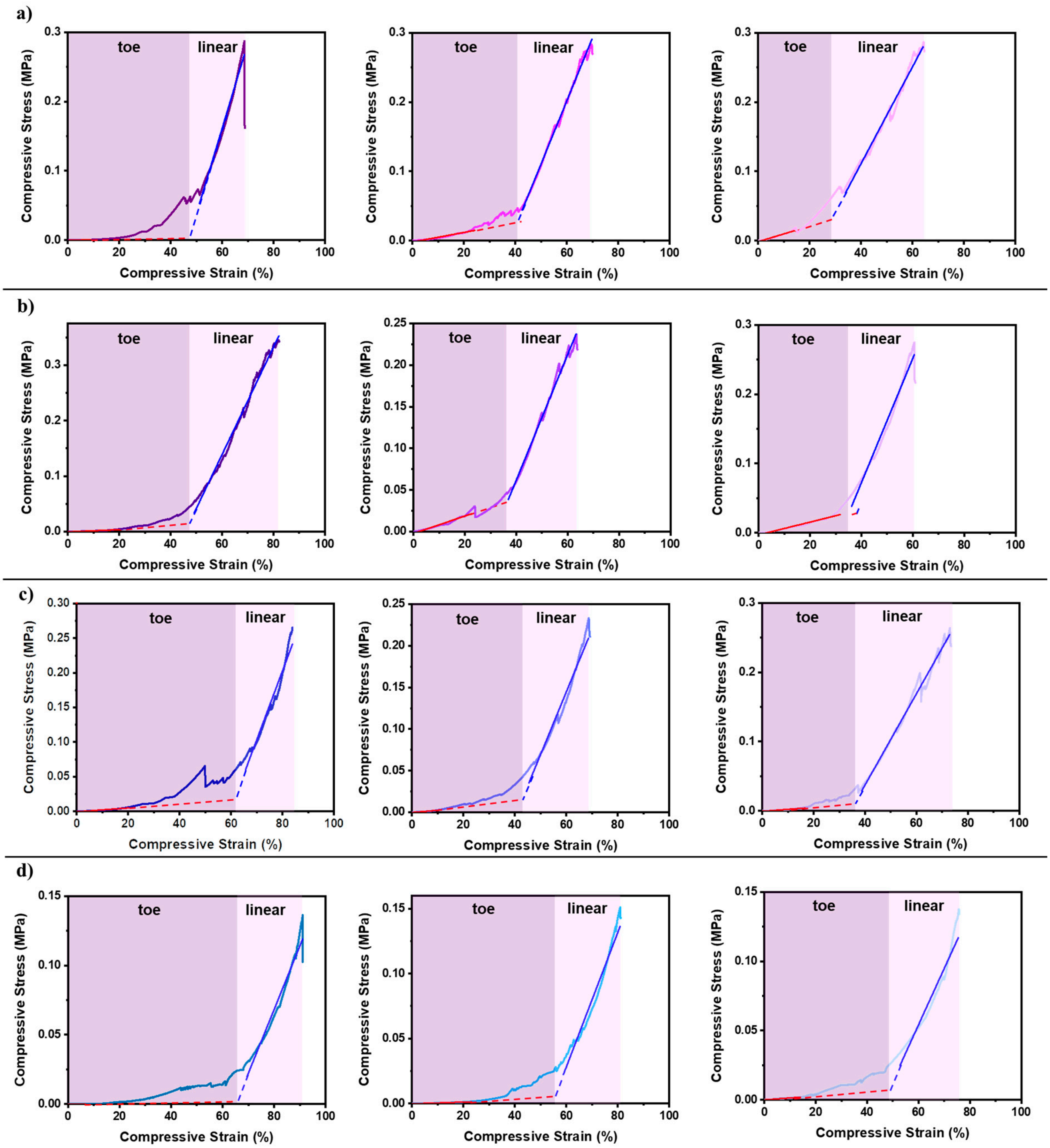

Figure S4. Stress–strain curves obtained from three independent replicates for all hydrogel formulations under uniaxial compression: (a) 0.33:0.66, (b) 0.50:0.50, (c) 0.66:0.33, and (d) 1.00:0.00. We distinguished between the toe modulus, which reflects the initial strain region commonly analyzed in soft matter mechanics, and the Young's modulus obtained from the subsequent linear (heal) region.

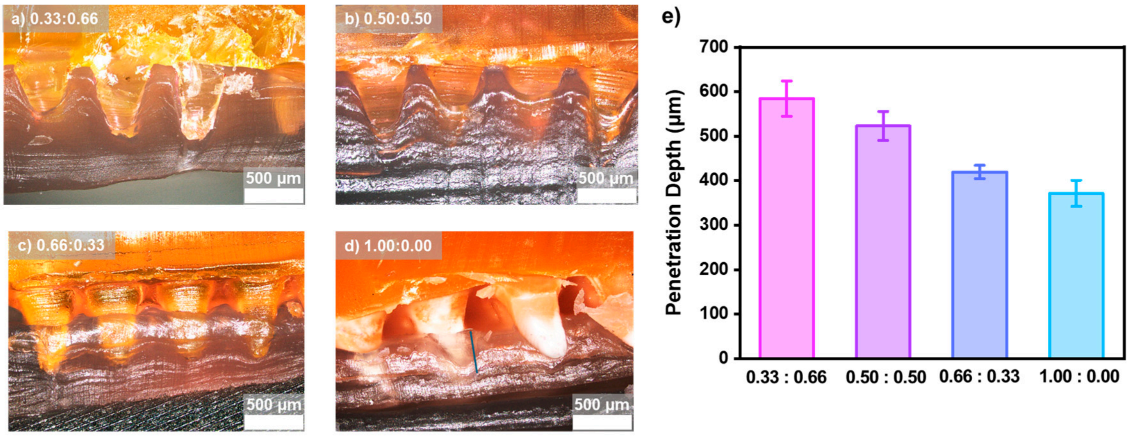

Figure S5. (a-d) Cross-sectional digital images of microneedles after penetration into artificial skin (parafilm) for formulations with PNIPAAm:AAm ratios of a) 0.33:0.66, b) 0.50:0.50, c) 0.66:0.33, and d) 1.00:0.00. e) Average penetration depth of microneedles into artificial skin (parafilm) for formulations with PNIPAAm:AAm ratios of 0.33:0.66, 0.50:0.50, 0.66:0.33, and 1.00:0.00.
